# Combinatorial Minigenome Systems for Emerging Banyangviruses Reveal Viral Reassortment Potential and Importance of a Protruding Nucleotide in Genome “Panhandle” for Promoter Activity and Reassortment

**DOI:** 10.3389/fmicb.2020.00599

**Published:** 2020-04-08

**Authors:** Fuli Ren, Min Zhou, Fei Deng, Hualin Wang, Yun-Jia Ning

**Affiliations:** ^1^State Key Laboratory of Virology, Wuhan Institute of Virology, Chinese Academy of Sciences, Wuhan, China; ^2^University of Chinese Academy of Sciences, Beijing, China; ^3^National Virus Resource Center, Wuhan Institute of Virology, Chinese Academy of Sciences, Wuhan, China; ^4^Center for Biosafety Mega-Science, Chinese Academy of Sciences, Wuhan, China

**Keywords:** banyangvirus, reverse genetics, minigenome, reassortment, untranslated region (UTR), genome panhandle, severe fever with thrombocytopenia syndrome virus (SFTSV), heartland virus (HRTV)

## Abstract

*Banyangvirus* is a new genus (*Phenuiviridae* family, *Bunyavirales* order) that comprises a group of emerging tick-borne viruses with severe fever with thrombocytopenia syndrome virus (SFTSV) and Heartland virus (HRTV) as virulent representatives. As segmented RNA viruses, bunyaviruses may have genome reassortment potential, increasing the concern about new life-threatening bunyavirus emergence. Using a series of combinatory minigenome reporter assays based on transfection and superinfection, we showed that replication machinery proteins of designated banyangviruses can recognize genomic untranslated regions (UTRs) of other banyangviruses and assemble heterogenous minigenomes into functional ribonucleoproteins (RNPs). Moreover, both heterogenous and heterozygous RNPs were efficiently packaged by viral glycoproteins into infectious virus-like particles, manifesting remarkable reassortment potential of banyangviruses. Meanwhile, UTR promoter strength of the three banyangvirus segments appeared to be M > L > S. Secondary structure analysis revealed a conservative non-basepairing protruding nucleotide in the terminal UTR panhandles of M and L (but not S) segments of all banyangviruses and some related phleboviruses (*Phlebovirus* genus). Furthermore, not only a conserved panhandle region but also the protruding nucleotide proved important for UTR function. Removal of the protruding nucleotide abated M and L UTR activities and compatibilities with heterogenous viral proteins, and introduction of a protruding nucleotide into S panhandle, conversely, enhanced UTR promoter strength and compatibility, revealing the significance of the protruding nucleotide as a new signature of the genomic panhandle structure in both UTR activity and reassortment potential. The study demonstrates not only banyangvirus reassortment potential but also the notable role of the protruding nucleotide in UTR function and reassortment, providing clues to viral evolution and replication mechanisms and perhaps benefiting disease control and prevention in the future.

## Introduction

Banyangviruses are a group of emerging tick-borne viruses (ICTV, 2018; Abudurexiti et al., 2019). These viruses were previously listed as the members of *Phlebovirus* genus (*Phenuiviridae* family, *Bunyavirales* order), but now are classified into *Banyangvirus* genus of the same family by International Committee on Taxonomy of Viruses (ICTV) (ICTV, 2018). Severe fever with thrombocytopenia syndrome virus (SFTSV) and Heartland virus (HRTV) are both emerging virulent representatives of *Banyangvirus* (ICTV, 2018; Abudurexiti et al., 2019). SFTSV was first identified in China in 2009 and then also found in the neighboring countries (Yu et al., 2011; Takahashi et al., 2014; Kim et al., 2018; Tran et al., 2019); HRTV disease cases were first reported in Missouri in 2012 and to date, have been discovered from 10 states in the Midwestern and southern United States (McMullan et al., 2012; Center for Disease Control and Prevention [CDC], 2018). Although SFTSV and HRTV are geographically isolated, they both can spread by tick bites and cause similar clinical symptoms in patients including severe fever, thrombocytopenia, leukocytopenia, gastrointestinal symptoms, hemorrhagic signs, and multiorgan dysfunction, associated with high case fatality rates (Yu et al., 2011; McMullan et al., 2012; Muehlenbachs et al., 2014; Center for Disease Control and Prevention [CDC], 2018). Following SFTSV and HRTV, Guertu virus (GTV) which was isolated from *Dermacentor nuttalli* ticks in China in 2014 by our research group is the third representative species of *Banyangvirus* listed by ICTV (Shen et al., 2018). Animal infection experiments demonstrated that GTV infection could result in pathological lesions similar to those caused by SFTSV (Liu et al., 2014; Matsuno et al., 2017; Shen et al., 2018). There are still several other neglected banyangviruses discovered throughout the world and associated with febrile or uncharacterized illnesses (Kokernot et al., 1969; Dilcher et al., 2012; Matsuno et al., 2013, 2015; Swei et al., 2013; Mourya et al., 2014; Wang et al., 2014). Although banyangviruses have posed severe threats to worldwide human health, there are no licensed vaccines or specific antivirals available currently.

Similar to phleboviruses, banyangviruses have negative-stranded tripartite RNA genomes, termed large (L) segment, medium (M) segment, and small (S) segment. L and M segments respectively encode viral RNA-dependent RNA polymerase (i.e., L protein) and the glycoprotein (GP) in a negative orientation; S segment encodes the nucleocapsid protein (N) and non-structural protein (NSs) with an ambisense polarity. The three genome segments of bunyaviruses all have 5′ and 3′ untranslated regions (UTRs) flanking the protein-coding open reading frames (ORFs). The UTRs function as promoters essential for ribonucleoprotein (RNP) packaging, replication, and transcription but vary in base sequence and length among different segments and viruses (Flick et al., 2004; Kohl et al., 2006; Terasaki et al., 2011). Despite the variance in sequence and length, the terminal nucleotides of 5′ and 3′ UTRs are highly genus-specific and significantly conserved on the three segments and moreover these nucleotides are inversely complementary facilitating the formation of a so-called double-stranded “panhandle” structure of each genome segment (Hewlett et al., 1977; Mir and Panganiban, 2006). Previous studies on some noted bunyaviruses have shown that the complementarity of the UTR panhandle is important for segment promoter activity (Barr and Wertz, 2004; Flick et al., 2004; Kohl et al., 2004; Mir and Panganiban, 2006). However, banyangvirus UTRs have not been characterized and moreover, other sequence or structure features of bunyavirus UTRs important for promoter activity still remain to be identified.

Because of genome segmentation and homology of the structural proteins and UTRs of genetically closely related bunyaviruses, their coinfections in host cells may result in genome segment reassortment and hence generation of new viruses packaging genome segments from different (parental) viruses (Briese et al., 2013). Progeny viruses that arise from reassortment may exhibit changed antigenicity, host tropism, transmissibility, and pathogenicity, leading to significant threats to the public health. Reassortment of the genome segments is likely an important mechanism for evolution of bunyavirus, similar to other segmented viruses like influenza virus (Briese et al., 2013; Lowen, 2017, 2018). Ushijima et al. (1981) found the phenomenon of potential natural reassortment for bunyaviruses, and thereafter, possible inter-specific and intra-specific natural reassortants of bunyaviruses have been reported continually, based on sequencing and phylogenetic analysis (Urquidi and Bishop, 1992; Sall et al., 1999; Reese et al., 2008; Yanase et al., 2010; Webster et al., 2011; Yanase et al., 2012; Fu et al., 2016; Shi et al., 2017; Calzolari et al., 2018; Coupeau et al., 2019). Interestingly, intra-specific natural reassortment of different genotypes appears to be involved in SFTSV evolution as well (Ding et al., 2013; Fu et al., 2016; Shi et al., 2017); however, whether inter-specific and even inter-genus reassortment could happen for banyangviruses remains unclear.

Minigenome system is a powerful reverse genetic tool for investigation of both viral UTR activity and reassortment potential in safer experimental settings. In the present study, we established a new RNA polymerase I (pol I)-based minigenome system for SFTSV. Using combinatorial minigenome reporter systems with transfection, superinfection, and infectious virus-like particle (iVLP) assays, we then investigated the reassortment potential of SFTSV, HRTV, and other related viruses and characterized the UTR activity of banyangviruses. Our findings strongly support that the genome segments of these viruses have notable reassortment potential. Meanwhile, we demonstrated that a conservative non-basepairing protruding nucleotide in banyangvirus genome panhandle is important for the optimal UTR activity and a single-site mutation (deletion or introduction) at this protruding nucleotide could significantly change the UTR activity and reassortment potential.

## Materials and Methods

### Cells and Viruses

BHK-21 (baby hamster kidney) cells, HEK293T (human embryonic kidney) cells, and Vero (African green monkey kidney) cells were grown in Dulbecco’s modified Eagle’s medium (DMEM, GIBCO) supplemented with 10% fetal bovine serum (FBS) at 37°C with 5% CO_2_. SFTSV (WCH97 strain) and HRTV (MO-4 strain) were grown in Vero cells and handled in a biosafety level 3 laboratory as previously described (Ning et al., 2014, 2015, 2019; Feng et al., 2019).

### Plasmids

To generate pol I-driven minigenome transcription plasmids, pRF42 which contains murine pol I promoter and terminator was used as the backbone vector (Flick and Pettersson, 2001). For construction of wild type or mutated UTR-containing minigenome reporters, firefly luc or EGFP reporter genes flanked by wild type or mutated viral UTR sequences were amplified by PCR and cloned into pRF42 in antisense orientation using restriction-free clone method with the In-Fusion HD Clone kit (Clontech). Along with the empty pRF42, pRF42-luc or pRF42-EGFP which respectively contains the antisense reporter genes fluc or EGFP without any UTR sequences flanked were used as controls.

The helper plasmids of minigenome reporter system which encode SFTSV N (pCAG-SV-N) or L protein (pCAG-SV-L) and the expression plasmid encoding HRTV GP (pCAG-HV-GP) were constructed by cloning the corresponding cDNA fragments into expression vector pCAGGSP7 with double restriction enzyme (*Kpn*I and *Not*I) digestion and DNA ligation. GenBank accession numbers of the viral segment reference sequences involved in the cloning of this study are as follows: JQ341188.1 (SFTSV L), JQ341189.1 (SFTSV M), JQ341190.1 (SFTSV S), JX005846.1 (HRTV L), JX005844.1 (HRTV M), JX005842.1 (HRTV S), KT328593.1 (GTV L), KT328592.1 (GTV M); KT328591.1 (GTV S), NC_001925.1 (BUNV L), NC_001926.1 (BUNV M), NC_001927.1 (BUNV, S), KM114246.1 (UUKV L), MG969385.1 (UUKV, M), DQ375417.1 (RVFV L), and DQ380213.1 (RVFV M). All the cloning constructs were confirmed by sequencing.

### Transfection and Minigenome Reporter Assays

In minigenome luc reporter assays based on transfection, 80% confluent BHK-21 cells cultured in 12-well plates were co-transfected with 1 μg indicated minigenome transcription plasmid, 500 ng L expression plasmid (pCAG-SV-L), 500 ng N expression plasmid (pCAG-SV-N), and 10 ng *Renilla* luc control plasmid (pRL-TK) per well using Lipofectamine 3000 reagent (Invitrogen) by following the manufacturer’s instructions. In transfection assays, the DNA-liposome complexes were removed after 6-h incubation, followed by careful washing and then incubation of cells with fresh medium for indicated time. Moreover, for control transfection groups, the total amount of DNA was kept constant by the addition of corresponding control plasmids. For the EGFP system, the minigenome-luc reporter plasmid was replaced by the corresponding minigenome-EGFP plasmid, and meanwhile the control plasmid pRL-TK was omitted. Forty-eight hours post-transfection, cells were delivered to luc activity measurement with a dual-luciferase reporter (DLR) kit (Promega) and relative luciferase activities (Rel. Luc. Act.) were then obtained by normalizing firefly luc activities to *Renilla* luc activities as described previously (Ning et al., 2014, 2015, 2017). Note that *Renilla* luc activities were comparable across experimental groups in the same DLR assays in our study, indicating constant transfection efficiency (Supplementary Figure S1A and data not shown). In the EGFP reporter system, EGFP expression was visualized under fluorescence microscope.

### Infectious Virus-Like Particle (iVLP) Assays

Infectious virus-like particle (iVLP) assays established in this study were used to investigate functional packaging of RNP by GP as described previously for Crimean-Congo Hemorrhagic Fever Virus (CCHFV) (Devignot et al., 2015) and UUKV (Overby et al., 2006). Firstly, BHK-21 cells were co-transfected with 1 μg indicated minigenome transcription plasmid, 500 ng pCAG-SV-L, 500 ng pCAG-SV-N, and 1 μg pCAG-HV-GP (or the empty vector) in 12-well cell culture plates. Forty-eight hours post-transfection, the supernatants were collected and then used to infect HEK293T which had been transfected with 1 μg pCAG-SV-L, 1 μg pCAG-SV-N, and 1 μg pCAG-HV-Gn/Gc in 6-well plates 12 h before the infection with iVLPs. In luc-based reporter system, 10 ng pRL-TK was also added in the DNA mixtures as an internal control for transfection efficiency. Forty-eight hpi, the HEK293T cells was delivered to EGFP expression detection or luc activity measurements as described above.

### Superinfection and Superinfection-Based iVLP Assays

To further confirm the results obtained from transient expression of viral structural proteins (including the replication proteins L and N and the envelop protein GP) by transfection, superinfections of BHK-21 cells with authentic viruses (SFTSV or HRTV) at an MOI of 3 were also conducted 12 h after transfection with 2 μg indicated minigenome transcription plasmid and 10 ng pRL-TK. Forty-eight hours post-superinfection, the cells were subjected to measurements of luc activities. Meanwhile, the supernatants were also collected to further infect fresh HEK293T cells and at 48 hpi, cells were delivered to luc activity detection.

### Statistical Analysis

Statistical analyses were conducted by GraphPad Prism 5 using Student’s *t-*test or one-way analysis of variance (ANOVA) method. All results were presented as mean ± SEM. *P-*value of <0.05 was considered statistically significant.

## Results

### Development of pol I-Based SFTSV Minigenome System

M and S segment minigenomes of SFTSV driven by bacteriophage T7 RNA polymerase (T7 pol) has been described previously (Brennan et al., 2015, 2017). However, T7 pol system requires specific cell lines which stably and ectopically express high levels of bacteriophage T7 pol and may lead to leaking expression (Lowen et al., 2004; Ikegami et al., 2006). Moreover, the transcription products from T7 pol are 5′-triphosphorylated RNAs which can trigger strong innate immune and inflammatory responses, remarkably changing cell states and strengthening cellular resistance to virus infection and replication (Hornung et al., 2006; Plumet et al., 2007). In comparison, RNA pol I-based system utilizing constitutively expressed enzyme in mammalian cells does not have these shortcomings and might be used as an alternative. Thus, we here exploited RNA pol I system for banyangvirus reverse genetic studies and firstly, murine pol I-driven minigenome systems for all the three segments of SFTSV were developed.

**FIGURE 1:**
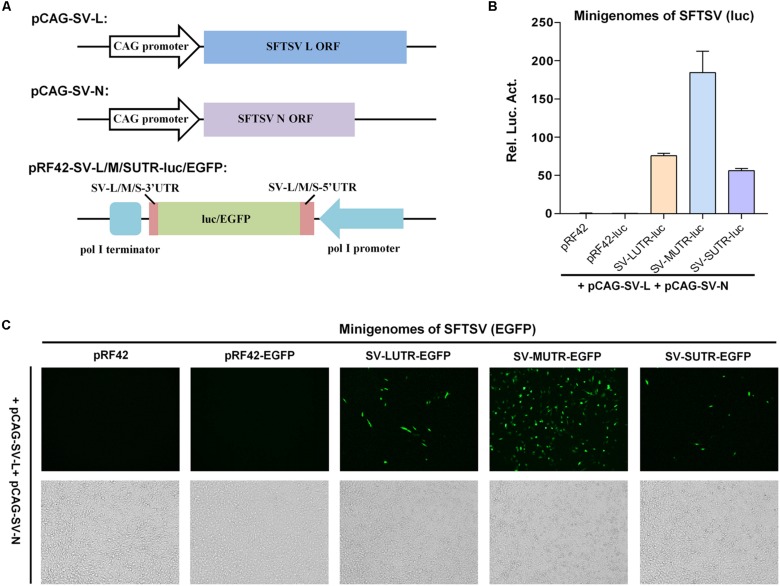
Establishment of pol I-driven SFTSV minigenome systems. **(A)** Schematic representation of the SFTSV minigenome system constructs. Expression plasmids for SFTSV L protein (pCAG-SV-L) or N (pCAG-SV-N) were generated by inserting the ORF sequences of L and N into pCAGGSP7. SFTSV L, M, or S-based minigenome transcription plasmids were constructed by cloning antisense-oriented reporter genes (firefly luc or EGFP) flanked by viral genomic UTRs into pol I-driven transcription vector pRF42. After transfection into cells, the pol I cassettes could allow generation of minigenome RNAs (viral genome segment analogs). When L and N can recognize the viral UTRs as functional promoters and assemble together with the minigenome RNAs into RNPs, replication and transcription of these minigenomes can occur, thus leading to reporter gene expression. **(B)** SFTSV minigenome activities in luc reporter system. BHK-21 cells were transfected with the minigenome-luc transcription plasmids for SFTSV L (SV-LUTR-luc), M (SV-MUTR-luc), or S segments (SV-SUTR-luc), together with pCAG-SV-L, pCAG-SV-N and internal control pRL-TK. Meanwhile, pRF42 empty plasmid and pRF42-luc that contains antisense firefly luc sequence without viral UTRs were transfected as negative controls for minigenome transcription plasmids. Forty-eight hours post-transfection, luc activities were measured. Rel. Luc. Act., relative luciferase activity. Data are presented as mean ± standard error of mean (SEM), *n* = 3. **(C)** SFTSV minigenome activities within EGFP reporter system. Cells were transfected with L and N expression plasmids, along with the indicated SFTSV minigenome transcription plasmids or the controls, respectively. Forty-eight hours post-transfection, EGFP expression was visualized under fluorescence microscope.

As shown in Figure 1A, expression plasmids for SFTSV L and N (named pCAG-SV-L and pCAG-SV-N, respectively) were generated by cloning the corresponding viral protein ORFs into expression vector pCAGGSP7 under the control of CAG promoter. SFTSV L, M, and S segment-based minigenome reporter plasmids were constructed by cloning the antisense firefly luciferase (luc) or enhanced green fluorescent protein (EGFP) cassette flanked by the UTRs of SFTSV L, M, and S segments into pRF42 under the control of pol I promoter to allow the generation of viral genome-like RNA segments, i.e., minigenomes, after transfection into cells (Figure 1A). When L and N recognize the RNA UTRs and assemble with the minigenomes into functional RNPs, replication and transcription can occur and thus result in reporter gene expression.

In luc reporter system, BHK-21 cells were co-transfected with the SFTSV L and N expression plasmids (or the empty pCAGGSP7 vector as negative control) and the minigenome transcription plasmids, together with an internal control plasmid expressing *Renilla* luc (pRL-TK), followed by measurements of luc activities at 48 h post-transfection. While no noticeable difference was observed for *Renilla* luc activities across the experimental groups (Supplementary Figure S1A), suggesting comparable transfection efficiency, all of the three segment-based minigenomes could be efficiently recognized by the viral N and L proteins, leading to robust expression of firefly luc reporter gene (Figure 1B). Meanwhile, the relative reporter gene activity indicated possibly differential promoter strength of SFTSV genome segments as M > L > S (Figure 1B). In contrast, no evident reporter activation was detected in absence of N and L in our minigenome reporter assays (Supplementary Figure S1B and data not shown), suggesting the specific activation of the minigenome-luc reporter by N and L (but not leaky expression) and further validating the established minigenome system. Furthermore, pol I-based minigenome systems for SFTSV could also work in assays with EGFP as the reporter (Figure 1C). Moreover, the apparently strongest promoter activity of M UTR was observed in this EGFP reporter system (Figure 1C). Consistently, no visible EGFP signal was found in the control minigenome system without N and L (Supplementary Figure S1C).

### SFTSV N and L Proteins Can Interact With Heterogenous Minigenomes of HRTV (But Not Bunyamwera Bunyavirus) to Form Functional RNPs

**FIGURE 2:**
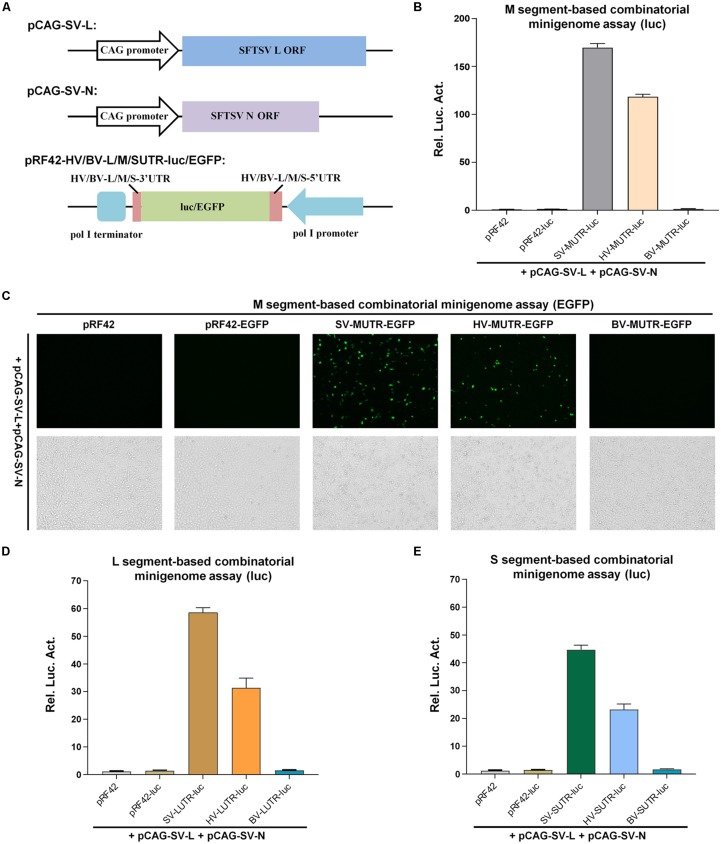
SFTSV N and L proteins can interact with heterogenous minigenomes derived from HRTV (but not BUNV) to form functional RNPs. **(A)** Schematic of the combinatorial minigenome system constructs. Minigenome reporter constructs for L, M, or S segments of HRTV (HV) or BUNV (BV) were generated similarly and termed as HV-M/L/SUTR-luc/EGFP or BV-M/L/SUTR-luc/EGFP, respectively. **(B)** M segment-based combinatorial minigenome activities within luc reporter system. BHK-21 cells were co-transfected with the indicated minigenome or control plasmids, along with pCAG-SV-L, pCAG-SV-N, and pRL-TK. Forty-eight hours post-transfection, luc activities were measured. Data are shown as mean ± SEM, *n* = 3. **(C)** M segment-based combinatorial minigenome activities within EGFP reporter system. Cells were co-transfected with the indicated minigenome or control plasmids, together with pCAG-SV-L and pCAG-SV-N, followed by EGFP expression visualization under fluorescence microscope at 48 h post-transfection. **(D,E)** SFTSV N and L proteins can also recognize HRTV L and S minigenomes to form functional RNPs. Cells were cotransfected with the indicated viral L **(D)** or S **(E)** minigenome transcription plasmids, together with the N and L protein expression plasmids and pRL-TK. Forty-eight hours post-transfection, cells were harvested for measurements of luc activities as described in **(B)**.

HRTV is another high-pathogenic banyangvirus discovered in United States (McMullan et al., 2012), following the identification of SFTSV. Considering the significant homology of the viruses, we hypothesized that heterozygous minigenome systems containing the RNA and protein components from different viruses may also work. Testing of combinatory minigenome reporter systems will benefit not only development of various indicator systems for virus infections but also understanding of virus replication mechanism and genome reassortment potential. For three-segmented banyangviruses, if they have genome reassortment potential, the prerequisite for reassortment is that the N and L protein must recognize heterogenous segments to form functional RNP complex. In the context of the successful establishment of pol I-driven SFTSV minigenome reporter system, we next developed a heterozygotic minigenome system containing HRTV segment-based minigenomes and SFTSV N and L proteins (Figure 2A) to test whether the replication machinery proteins of SFTSV can recognize the genome segments of HRTV.

Firstly, SFTSV L and N indeed could noticeably drive the heterogenous HRTV M segment-based minigenome activity to a large extent (69.8% of homozygous SFTSV M minigenome activity) as shown in luc reporter assay (Figure 2B), revealing the efficient assembly of functional heterozygous RNP. By contrast, M minigenome of Bunyamwera bunyavirus (BUNV) that is a protype bunyavirus (*Orthobunyavirus* genus, *Peribunyaviridae* family) (ICTV, 2018) distantly related to banyangviruses (included here as a control) could not be recognized (Figure 2B). Moreover, similar results were observed in minigenome system using EGFP as the reporter (Figure 2C). Next, luc reporter assays further showed that HRTV L and S segment-based minigenomes could interact with SFTSV L and N to form functional RNPs as well (Figures 2D,E), and the strength of HRTV minigenome activity driven by SFTSV L and N was also in the order M > L > S, similar to the findings in homozygous SFTSV minigenome system (Figures 1B, 2B,D,E). Together, these results suggest that SFTSV N and L proteins can recognize all the three heterogenous minigenomes from HRTV to form functional heterozygous RNPs.

### Both Heterogenous and Heterozygous Banyangvirus RNPs Can Be Packaged by GP Into iVLPs

**FIGURE 3:**
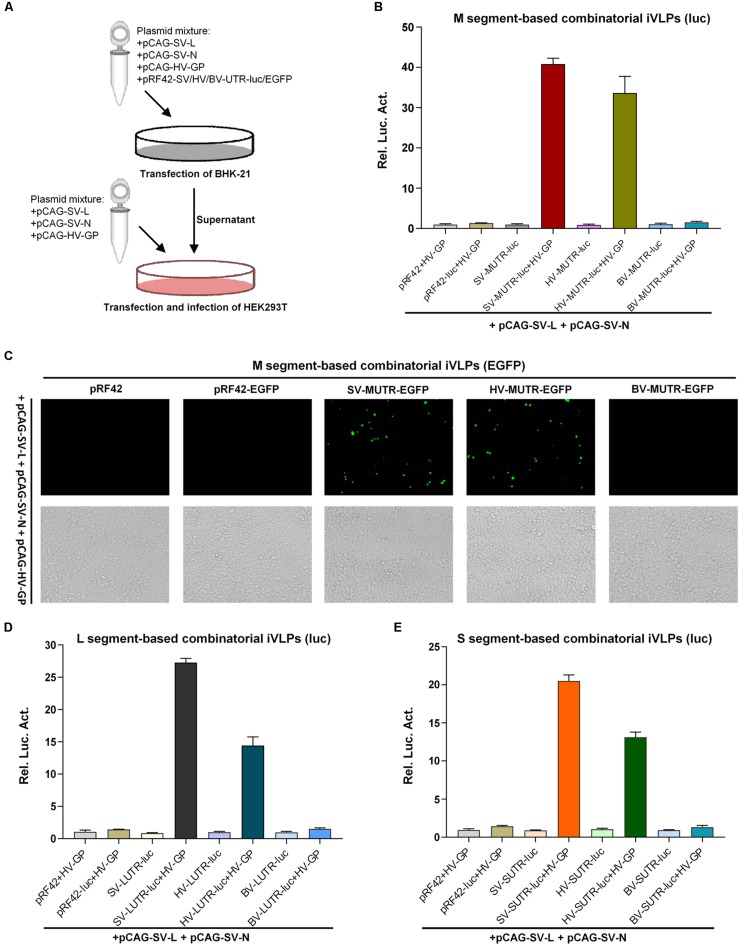
Banyangvirus GP can package combinatory RNPs into iVLPs. **(A)** Schematic of combinatory iVLP assays. Briefly, BHK-21 cells were cotransfected with the control or minigenome reporter plasmids based on L, M, or S segments of SFTSV (SV), HRTV (HV), or BUNV (BV), along with the indicated viral structural protein expression plasmids. In some groups, GP expression plasmid (pCAG-HV-GP) was replaced with the empty vector as control. Forty-eight hours post-transfection, supernatants of the cell cultures were collected and used to infected HEK293T cells which had been transfected with the viral structural protein expression plasmids 12 h before iVLP infection. In luc reporter system, pRL-TK was also included in transfection as an internal control. At 48 hpi, reporter gene expression was detected by enzyme activity analysis or fluorescence microscopy. **(B,C)** Packaging of M minigenome-containing RNPs by GP into iVLPs. iVLPs were generated by co-transfection of BHK-21 cells with the indicated viral M minigenome plasmids with luc **(B)** or EGFP **(C)** as the reporter gene and the N and L protein expression plasmids, along with HRTV GP expression plasmid or the corresponding empty vector as control. Then, supernatants potentially containing iVLPs were collected and used to infected HEK293T transiently expressing viral structural proteins, followed by luc activity **(B)** or EGFP expression **(C)** detection as described in **(A)**. **(D,E)** Packaging of L or S minigenome-based RNPs by GP into iVLPs. Viral L **(D)** or S **(E)** minigenome-based iVLP assays were performed as in **(B)**. Graphs show mean ± SEM, *n* = 3.

In addition to formation of functional heterozygous RNPs, GP-mediated packaging of heterogenous and heterozygous RNPs into new infectious virus particles is the next requirement for accomplishment of reassortment event. Thus, we next investigated the capacity of banyangvirus GP to package heterogenous and heterozygous RNPs generated above into iVLPs. Schematic of combinatory iVLPs generation using HRTV GP as the envelope protein was shown in Figure 3A. Briefly, BHK-21 cells were co-transfected with pCAG-SV-L, pCAG-SV-N, and the indicated minigenome plasmids, together with HRTV GP expression plasmid or the vector control. At 48 h post-transfection, the supernatants potentially containing iVLPs were collected to infect HEK293T cells expressing the viral structural proteins. At 48 h post-infection (hpi), luc activities or EGFP expression in HEK293T cells were analyzed respectively. Firstly, luc reporter assays show that substantial firefly luc activation could be specifically detected in SFTSV and HRTV (but not BUNV) M segment-based minigenome groups in the presence of HRTV GP (Figure 3B), suggesting that HRTV GP can package not only heterogenous SFTSV RNP containing M minigenome RNA, L and N of SFTSV but also heterozygous RNP consisting of HRTV M minigenome RNA and SFTSV L and N. Similar results were obtained in iVLP assays with EGFP as the reporter (Figure 3C). Furthermore, combinatory RNPs with L or S minigenomes of SFTSV and HRTV all could be packaged by HRTV GP into iVLPs that then mediated notable reporter gene expression in HEK293T cells (Figures 3D,E). Consistent with the results of minigenome reporter assays based on transfection in Figures 1, 2, these iVLP-mediated minigenome reporter assays also indicate the stronger activities of M and L minigenomes.

### Superinfection Assays Confirmed the Significant Reassortment Potential and Differential UTR Activities of Banyangvirus Genomes

**FIGURE 4:**
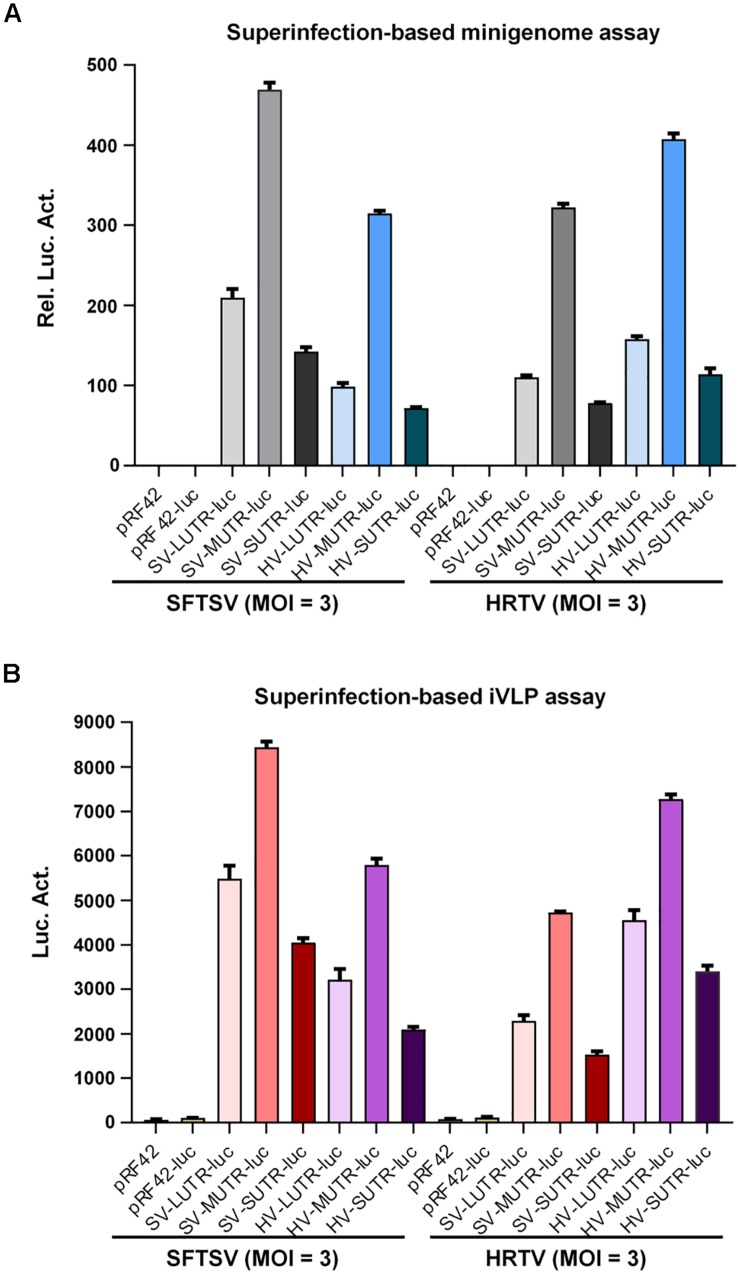
Superinfection-based minigenome and iVLP assays. BHK-21 cells were transfected with the indicated control or minigenome plasmids based on L, M, or S segments of SFTSV or HRTV, together with pRL-TK. Twelve hours post-transfection, cells were respectively superinfected with SFTSV or HRTV (MOI = 3). Forty-eight hours post-superinfection, cells were delivered to luc activity measurements and the relative luc activities (Rel. Luc. Act.) were shown in **(A)**. See also Table 1. Meanwhile, supernatants of the superinfected BHK-21 cell cultures were collected and used to infect fresh HEK293T cells, followed by firefly luc activity (Luc. Act.) detection at 48 hpi **(B)**. Data are presented as mean ± SEM, *n* = 3.

To further corroborate banyangvirus reassortment potential, superinfection assays with SFTSV and HRTV were conducted to respectively analyze SFTSV or HRTV L, M, and S segment-based minigenome activities. BHK-21 cells transfected with SFTSV or HRTV minigenome plasmids were superinfected with the cognate or heterogenous viruses at 12 h post-transfection. At 48 h post-superinfection, cells were delivered to luc activity measurement and meanwhile the supernatants were also collected for infection of fresh HEK293T cells, followed by further luc activity detection. As shown in Figure 4A, SFTSV and HRTV both could drive their cognate minigenome activities by providing the replication machinery proteins (L and N) in BHK-21 cells and consistent with the minigenome reporter assays based on transfection (Figures 1, 2) or iVLPs (Figure 3), the segment promoter strengths driven by their cognate viruses, SFTSV or HRTV, both are likely M > L > S (Figure 4A). Interestingly, superinfections with the two high-pathogenic viruses could also lead to remarkable reporter activities of heterogenous minigenomes (Figure 4A), confirming the functional formation of high-active heterozygous RNPs. To further show the efficient cross-recognition activities, relative minigenome activities of HRTV or SFTSV M, L, and S segments driven by heterogenous virus superinfections were respectively calculated and summarized in Table 1. Further, minigenome activities stimulated by heterogenous viruses are also M > L > S, in accordance with those driven by cognate viruses (Figure 4A). Then, luc reporter assays with HEK293T cells revealed that SFTSV or HRTV superinfection of BHK21 expressing cognate or heterogenous minigenome RNAs resulted in package of various combinatory iVLPs carrying cognate or heterogenous minigenomes and structural proteins which could further infect HEK293T cells and drive high levels of minigenome reporter activities (Figure 4B). Collectively, the efficient package of combinatory functional RNPs and iVLPs as indicated by transfection, iVLP, and superinfection-based minigenome assays confirms banyangvirus remarkable reassortment potential.

**TABLE 1:** Relative minigenome activities of banyangvirus M, L, or S segments driven by cognate or heterogenous virus superinfections.

Minigenome^*b*^	Relative minigenome activity^*a*^ driven by superinfections	
	HRTV superinfection	SFTSV superinfection
HV-MUTR-luc	100%	77.2%
HV-LUTR-luc	100%	62.4%
HV-SUTR-luc	100%	63.2%
SV-MUTR-luc	68.7%	100%
SV-LUTR-luc	52.5%	100%
SV-SUTR-luc	54.9%	100%

*^a^Relative activities of the indicated viral minigenomes driven by heterogenous virus superinfection were calculated by normalizing their relative luc activity means obtained from Figure 4A to those driven by cognate virus superinfection (Figure 4A); correspondingly, relative minigenome activities driven by cognate virus superinfection were set to 100%. ^b^HV-MUTR-luc, HV-LUTR-luc, and HV-SUTR-luc, minigenomes based on HRTV M, L, and S segments, respectively; SV-MUTR-luc, SV-LUTR-luc, and SV-SUTR-luc, minigenomes based on SFTSV M, L, and S segments, respectively.*

### M and L Segments of Banyangviruses and Some Phleboviruses Share a Conserved Non-basepairing Protruding Nucleotide in the Double-Stranded Panhandle Structure

**FIGURE 5:**
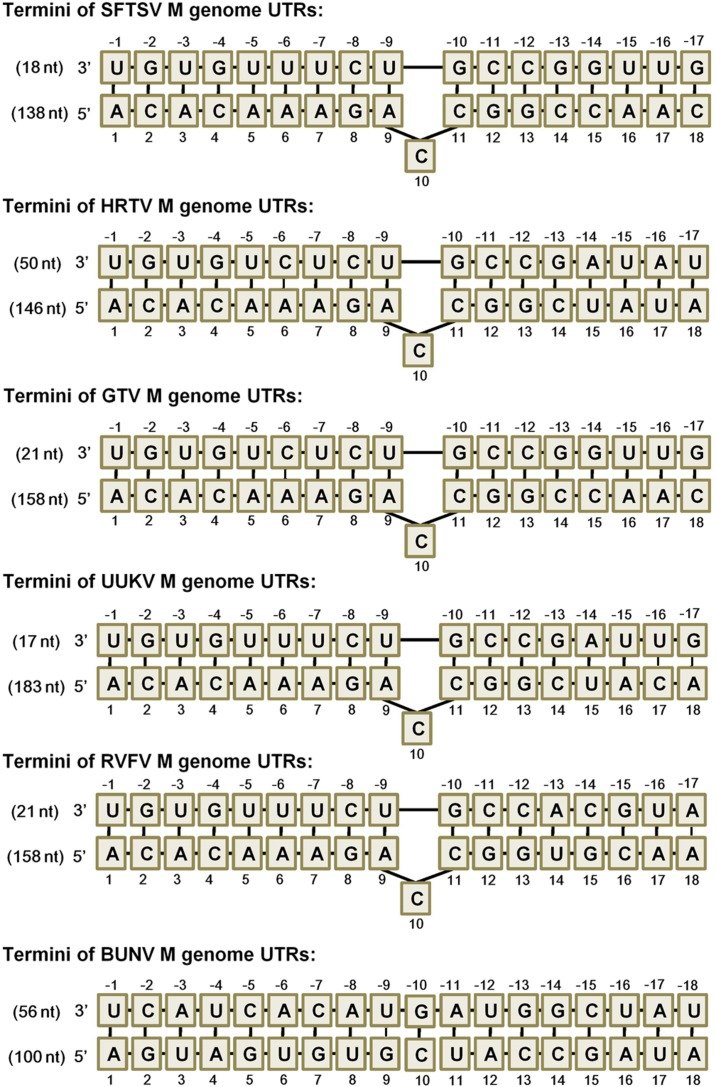
Predicted terminal double-stranded panhandles of M genome RNAs. UTR sequences of M genomes of banyangviruses, phleboviruses, and the protype bunyavirus BUNV were respectively analyzed with M-fold program. The predicted double-stranded panhandle structures of banyangviruses are similar to those of phleboviruses and a region formed by 5′ terminal 13 nucleotides and 3′ terminal 12 nucleotides of the genome RNAs was particularly conserved among all banyangviruses and some related phleboviruses (but not BUNV). Watson-Crick base pairs in the panhandle structures are indicated with bold lines and non-canonical paired bases are marked with fine lines. In addition to the paired nucleotides, a non-basepairing protruding nucleotide C at the position 10 of 5′ termini (for genome RNAs) in the context of double-stranded panhandles is also conserved among all known banyangviruses and some related phleboviruses (including the representative UUKV and RVFV), but not BUNV. The protruding nucleotide-harboring panhandle regions of only the representative virus members are shown. Nucleotide positions of 5′ and 3′ UTRs are respectively defined with positive and negative numbers as indicated. The total lengths of 5′ and 3′ UTRs are indicated in parentheses (nt, nucleotides).

Previous studies on some noted bunyaviruses have shown that the terminal inverse complementarity of bunyavirus UTRs allowing panhandle formation is critical for promoter activity (Flick et al., 2002; Barr and Wertz, 2004; Kohl et al., 2004; Mir and Panganiban, 2006). Although the terminal complementary sequences of banyangvirus M, L, and S UTRs are highly similar, the differential minigenome activities of the three segments (M > L > S) as shown above suggest that some detailed differences in the complementary region or beyond can also affect the promoter strength. To gain further insights into the important characteristics of the panhandle structures for UTR promoter activity, the secondary structures of M UTRs of banyangviruses and related phleboviruses were firstly predicted using M-fold program as described previously (Khromykh et al., 2001; Zuker, 2003; Pontrelli et al., 2004). The 5′ and 3′ UTRs of banyangvirus M segments could form double-stranded panhandles by the inversely complementary sequences, similar to those of phleboviruses and in particular, a terminal region of the panhandles formed by 5′ terminal 13 nucleotides and 3′ terminal 12 nucleotides (for genome RNAs) is highly conserved among all banyangviruses and some related phleboviruses (but not BUNV) (Figure 5 and data not shown). Intriguingly, we also noticed that the 10^th^ nucleotide C (10C) of 5′ UTRs for genome RNAs (or -10G of 3′ UTRs in antigenomes) was predicted as a non-basepairing “redundant” nucleotide in the context of the terminal complementary regions (Figure 5). This protruding nucleotide in the double-stranded panhandle structures is conserved for all known banyangviruses and even some phleboviruses including Uukuniemi virus (UUKV) and Rift Valley fever virus (RVFV) (Figure 5). The complementary sequences with the protruding nucleotide of some representative viruses are shown in Figure 5 and the terminal panhandle sequences of BUNV without a protruding nucleotide at position 10 are also included in comparison. Further, similar analysis of L UTRs suggests that the protruding 10C is conservative in L genome panhandle structures of all banyangviruses and some related phleboviruses as well (data not shown). In contrast, there is not a conservative protruding nucleotide in the panhandles of S segments that have the weakest promoter activities, in comparison with M and L. We thus hypothesized that the protruding nucleotide may play a role in the UTR activity of banyangvirus genomes.

### The Conserved Terminal Panhandle Region With the Protruding Nucleotide Is Crucial for Banyangvirus UTR Activity

**FIGURE 6:**
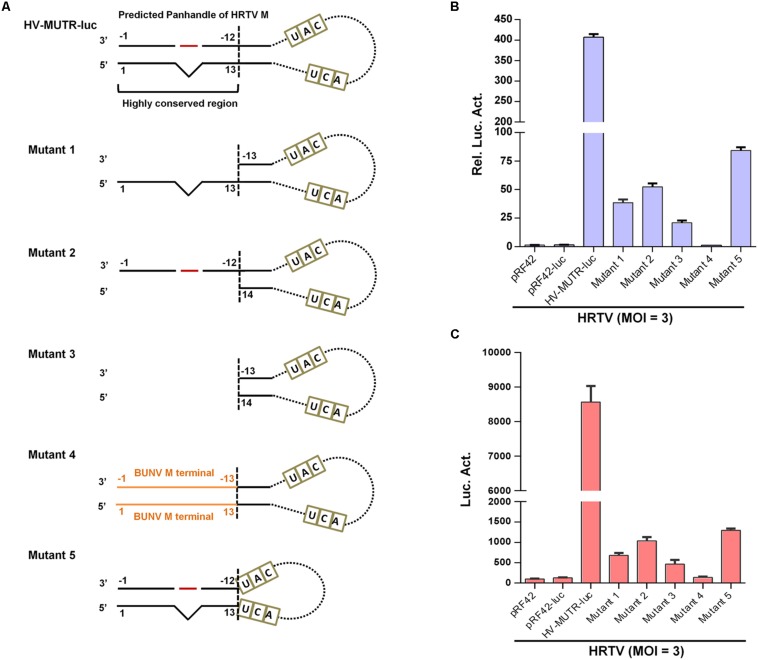
Role of the conservative terminal panhandle region with the protruding nucleotide in UTR activity. **(A)** Schematic of HRTV M minigenome and its mutants with the conservative terminal panhandle disrupted. For HRTV M genome RNA, 5′ and 3′ UTRs contain 146 and 50 nucleotides, respectively. Nucleotides UAC and UCA represent the gene start and stop codons (antisense) in M genome, respectively. The highly conserved terminal region of the double-stranded panhandles of banyangviruses and some related phleboviruses consists of 5′ terminal 13 and 3′ 12 nucleotides. See also Figure 5. For mutant 1, 12 nucleotides of 3′ terminal were deleted; for mutant 2, 13 nucleotides of 5′ terminal were deleted; for mutant 3, 12 nucleotides of 3′ terminal and 13 nucleotides of 5′ terminal were both deleted; for mutant 4, the highly conserved region was replaced by the terminal panhandle (13 pairs of nucleotides) of BUNV; for mutant 5, only the highly conserved region was retained as the 5′ and 3′ UTRs. **(B,C)** BHK-21 cells were transfected with the indicated wild type or mutant minigenome plasmids (with luc as reporter) or the controls, together with pRL-TK. At 12 h post-transfection, cells were superinfected with HRTV (MOI = 3), followed by luc activity measurements at 48 hpi **(B)**. Meanwhile, the supernatants of the superinfected BHK-21 cell cultures were harvested to infected fresh HEK293T cells, followed by further luc activity analyses **(C)**. Data are shown as mean ± SEM, *n* = 3.

To analyze the role of the conserved terminal panhandle structure with the protruding nucleotide in banyangvirus UTR activity, we examined the effects of fragment deletion or replacement of terminal sequences (Figure 6A) on minigenome activity by superinfection and iVLP assays. With HRTV M minigenome system as an experimental model, we demonstrated that deletion of the 5′ or/and 3′ terminal sequences (mutants 1, 2, and 3) largely depleted the minigenome activity in both superinfection- (Figure 6B) and iVLP-mediated reporter assays (Figure 6C), indicating the critical role of the region in UTR promoter activity. Moreover, replacement of the region by the corresponding terminal complementary sequences of BUNV (mutant 4) did not restore but instead abolished minigenome reporter activity (Figures 6B,C), suggesting the banyangvirus specific requirement of the terminal panhandle region. Furthermore, interestingly, a minigenome with only the conserved terminal panhandle region (mutant 5) facilitated a reduced but substantial expression of the reporter gene and moreover, its activity (mutant 4) is much stronger than that of mutant 3, although the latter contains longer UTR sequences at both 5′ and 3′ termini (Figures 6B,C). These results validate the important role of the identified terminal panhandle region with the protruding nucleotide in banyangvirus UTR activity.

### The Significant Role of the Protruding Nucleotide of Banyangvirus Terminal Panhandle in M and L UTR Activities

**FIGURE 7:**
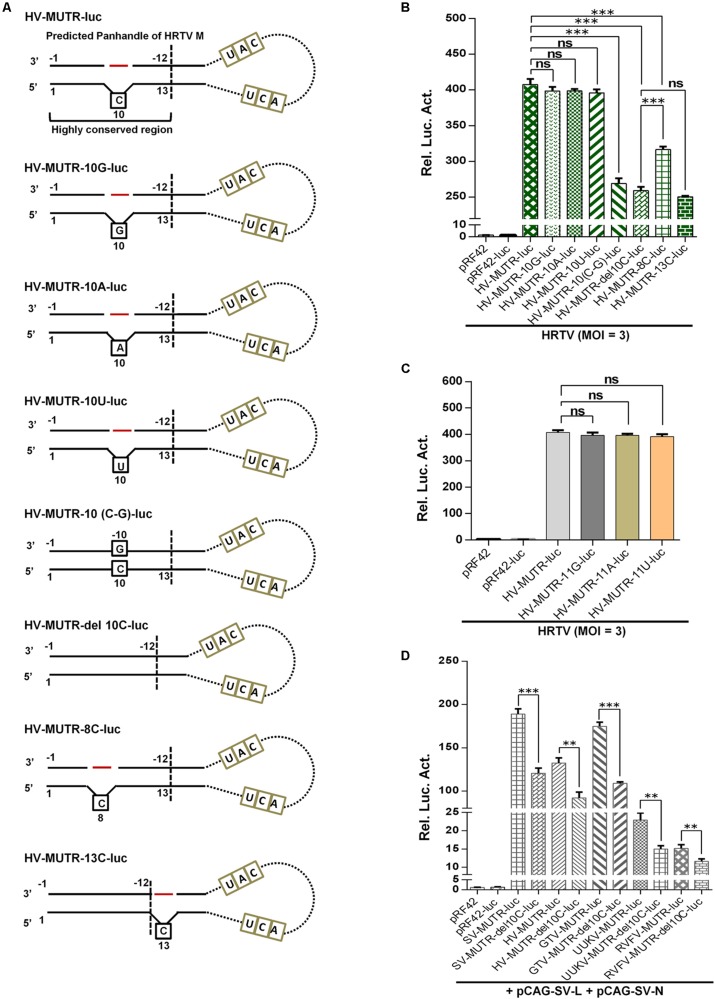
The conserved protruding nucleotide of the panhandle structure is important for M genome UTR activity. **(A)** Schematic of HRTV M minigenomes with the protruding nucleotide mutated. HV-MUTR-10G, HV-MUTR-10A, and HV-MUTR-10U refer to the mutants of which the protruding 10C in 5′ UTR was mutated to G, A, and U, respectively. HV-MUTR-10(C-G), the mutant with base-pairing C-G at positions 10 of 5′ UTR and –10 of 3′ UTR by inserting a G into 3′ terminal correspondingly. HV-MUTR-del10C, the mutant with the protruding 10C deleted. HV-MUTR-8C and HV-MUTR-13C, the mutants in which a C was respectively introduced at position 8 or 13 of 5′ UTR based on the mutant “HV-MUTR-del10C” by PCR. **(B)** BHK-21 cells transfected with the indicated control or minigenome luc plasmids and pRL-TK were superinfected with HRTV (MOI = 3) at 12 h post-transfection, followed by luc activity measurements at 48 hpi. **(C)** Mutants with the protruding 11C changed to G, A, or U were also similarly constructed. Superinfection-based minigenome reporter assays were conducted as in **(B)**. **(D)** Minigenome-luc plasmids for M segments of GTV (GV-MUTR-luc), UUKV (UV-MUTR-luc), and RVFV (RV-MUTR-luc) were similarly generated by cloning the viral M minigenome-luc chimeras into pRF42 and based on these wild type minigenomes, the corresponding mutants with the protruding 10C deleted were further constructed. BHK-21 cells were co-transfected with the indicated minigenome-luc plasmids or the controls, together with the SFTSV N and L expression plasmids and pRL-TK. At 48 h post-transfection, luc activities were measured. Data shown are mean ± SEM, *n* = 3. ^∗∗^*P* < 0.01; ^∗∗∗^*P* < 0.001; ns, non-significant.

Next, we continued to investigate whether the conservative protruding nucleotide flanked by complementary sequences of the terminal panhandle structure plays a role in banyangvirus UTR activities. As indicated by site-directed mutation analyses combined with superinfection and iVLP minigenome reporter assays, substitution of the protruding 10C by G, A, or U did not noticeably affect HRTV M UTR activity driven by HRTV superinfection (Figures 7A,B); however, removal of the protruding nucleotide by directly deleting 10C or inserting a base-pairing G in 3′ terminal (−10G) substantially reduced the minigenome activity (Figures 7A,B). Although 10C was predicted as the protruding nucleotide by the M-fold program, because there is a repeated C at position 11 of 5′ terminal (Figure 5A), this nucleotide (11C) may potentially replace 10C as the protruding one. Interestingly, substitution of 11C by G, A, or U which could render the nucleotide at position 11 protruding did not affect HRTV M UTR activity either (Figure 7C). These data suggest that the protruding nucleotide at position 10 (or 11) of 5′ terminal of genome panhandle likely plays a significant role in HRTV M UTR function. To further analyze whether the position of the protruding nucleotide is important, we meanwhile tested the effects of position changes of the protruding nucleotide on the UTR activity. Reporter assays showed that the minigenome activity of a mutant with introduction of a protruding 8C on the basis of 10C deletion was significantly restored, but still lower than that of the wild type (Figures 7A,B); further, in comparison with the mutation with 10C deleted, introduction of a protruding 13C that locates outside of the conserved terminal region of the panhandle did not restore the minigenome activity at all (Figures 7A,B), suggesting that the position of the protruding nucleotide also matters for the optimal HRTV M UTR activity. Similar results were also observed in minigenome reporter assays with iVLPs from the cell culture supernatants of the corresponding superinfections above (data not shown).

Then, the notable role of the protruding nucleotide in M UTR activity was validated for other banyangviruses, as shown in Figure 7D. As expected, M minigenome of GTV could be strongly triggered by SFTSV L and N to an extent stronger than that of HRTV and comparable with the homozygous SFTSV minigenome system, while the protruding nucleotide removal significantly reduced all the reporter activities whether for homozygous or for heterozygous minigenome systems (Figure 7D). In addition, intriguingly, SFTSV L and N could also drive M minigenome activities of UUKV and RVFV albeit to lesser extents and further, deletion of the protruding 10C of the phleboviruses impaired their M UTR activities driven by SFTSV protein machinery as well (Figure 7D). The data not only confirm the importance of the protruding nucleotide for M UTR function but also reflect various degrees of reassortment potential of SFTSV with the other tested viruses. Moreover, deletion of the protruding nucleotide may lead to reduction of the reassortment potential among these viruses.

**FIGURE 8:**
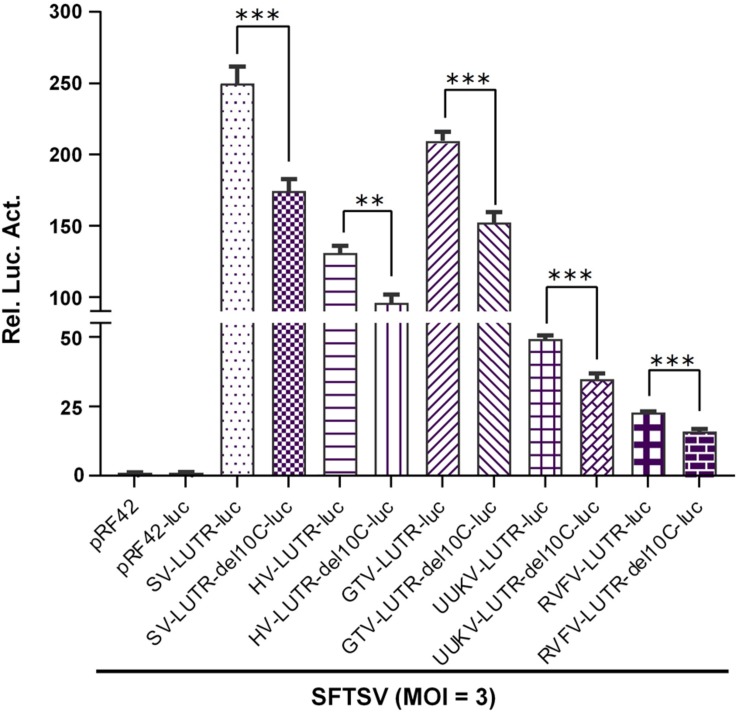
The conserved protruding nucleotide in L genome panhandles also plays a significant role in L UTR activity. The indicated wild type minigenome-luc plasmids based on viral L segments or the corresponding mutants with the protruding 10C deleted were transfected into BHK-21 cells. At 12 h post-transfection, cells were superinfected with SFTSV (MOI = 3), followed by luc activity measurements at 48 hpi. Data shown are mean ± SEM, *n* = 3. ^∗∗^*P* < 0.01; ^∗∗∗^*P* < 0.001.

As the protruding nucleotide is also conserved in L segment panhandles of banyangviruses as well as UUKV and RVFV, we thus further examined its role in L UTR activity. As shown in Figure 8, deletion of the protruding 10C significantly decreased L minigenome activities of all the tested banyangviruses (including SFTSV itself) and UUKV and RVFV driven by SFTSV superinfection as well, indicating the significant role of the protruding nucleotide in L UTR function and the possible effect of this nucleotide on viral reassortment potential.

### Analysis of the Protruding Nucleotide in the Context of Banyangvirus S Panhandle

**FIGURE 9:**
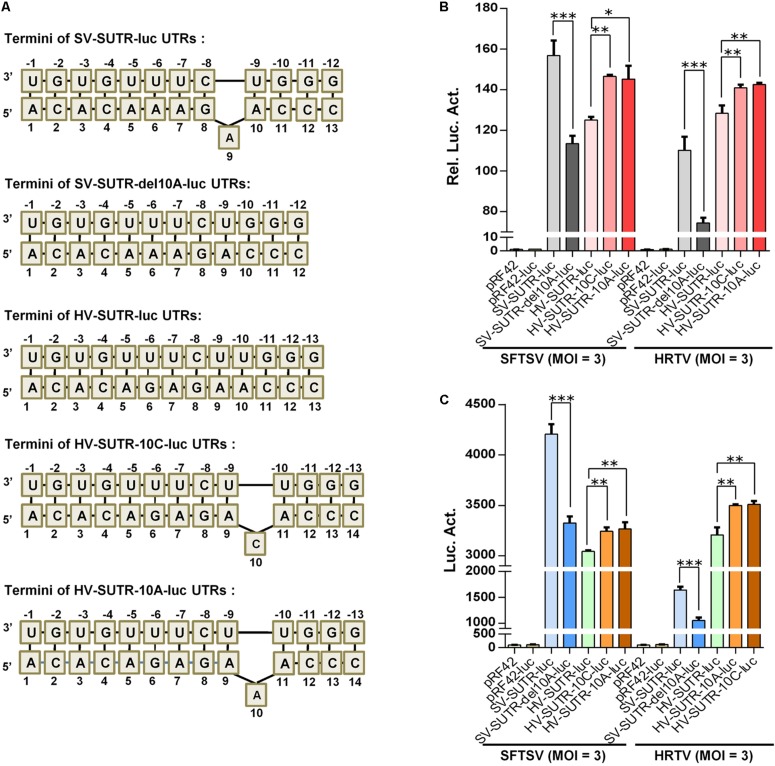
Functional analysis of the protruding nucleotide in the context of S panhandle terminal. **(A)** Schematic of UTR termini of S minigenomes and the mutants. The terminal panhandle region formed by wild type SFTSV S genome UTRs (SV-SUTR) contains a protruding A at position 9 or 10 of 5′ UTR, while there is no protruding nucleotide in the corresponding regions of other banyangviruses, including that formed by HRTV S genome UTRs (HV-SUTR). SV-SUTR-del10A, the SV-SUTR mutant of which the protruding 9A (or 10A) was deleted. HV-SUTR-10C, the HV-SUTR mutant in which a protruding C was introduced at position 10 of 5′ UTR. HV-SUTR-10A, the HV-SUTR mutant with a protruding 10A (or 9A or 11A) in HRTV S panhandle. **(B,C)** BHK-21 cells were transfected with the control vectors or the indicated minigenome-luc reporter plasmids based on S UTRs or their mutants, together with pRL-TK. Twelve hours post-transfection, cells were respectively superinfected with SFTSV or HRTV (MOI = 3) for 48 h, before luc activity analyses **(B)**. The supernatants of the superinfected cell cultures potentially containing iVLPs were also harvested for further infection of fresh HEK293T cells, followed by luc activity measurements **(C)**. Data are expressed as mean ± SEM, *n* = 3. ^∗^*P* < 0.05; ^∗∗^*P* < 0.01; ^∗∗∗^*P* < 0.001.

By structural predication and minigenome mutation analyses, we further found that the S panhandle terminal regions of some SFTSV strains contain a potential protruding 9/10A (Figure 9A, with WCH97 strain as an example) and removal of this nucleotide also reduced SFTSV S UTR activity actuated by cognate and heterogenous virus superinfections (Figures 9A,B). However, the protruding nucleotide is not conserved in S panhandles of either some other SFTSV strains or the other banyangviruses (Figure 9A and data not shown). On the basis of these observations, we next investigated whether introduction of a protruding nucleotide into the S panhandle terminal region of other banyangviruses without such a nucleotide originally (like that of HRTV shown in Figure 9A) can influence the viral UTR activity driven by the cognate or heterogenous viruses. Interestingly, the addition of a nucleotide C or A at position 10 that could respectively generate a protruding 10C or 10A (or 9/11A) significantly enhanced HRTV S UTR activity driven by both homogenous HRTV and heterogenous SFTSV superinfections (Figures 9A,B). Similar results were observed in iVLP-based minigenome reporter assays (Figure 9B). These findings not only support the notable role of the protruding nucleotide in banyangvirus UTR activity but also suggest that one single nucleotide mutation may increase homozygous and heterozygous RNP activities and hence viral replication and reassortment potential to some extent.

## Discussion

As an emerging group of bunyaviruses, *Banyangvirus* contains multiple medically important pathogens, posing a severe threat to public health (ICTV, 2018). Therein, SFTSV that can cause a severe hemorrhagic fever-like disease with a high case fatality rate of up to 30% was listed as one of the top priority pathogens for research and development by Word Health Organization [WHO] (2017). Better understanding of banyangvirus replication and evolution mechanisms will be beneficial for prevention and control of the life-threatening infectious diseases caused by these viruses. Apart from genome recombination and point mutations, reassortment is an additional powerful strategy underlying evolution of some segmented viruses and largely increases the risk of novel high-pathogenic virus emergences (Briese et al., 2013; Shi et al., 2017). In the present study, using combinatory minigenome reporter assays, we demonstrated that the structural components of banyangviruses, including the structural proteins and genome *cis*-acting elements (UTRs), exhibit significant functional compatibility, reflecting strong reassortment potential among these viruses. Furthermore, a conservative protruding nucleotide in the context of the terminal UTR complementary regions was identified as a new signature of genomic panhandle structures of banyangviruses and some phleboviruses, which likely plays significant roles in not only viral replication efficiency but also reassortment potential. These results support the possibilities that new virulent banyangviruses might be generated by reassortment and even a single nucleotide mutation might enhance the pathogenicity and reassortment potential. Future epidemiological investigations including isolation and sequence analysis of more virus strains from various animal and tick hosts may provide further evidence for these laboratory findings based on molecular biology.

Recently, Rezelj et al. (2019) reported UUKV, SFTSV, and HRTV M segment-based minigenome activities in the presence of heterogenous N and L, using T7 pol-driven minigenome system with transient transfection, and tested heterogenous GP packaging of homozygous L or S RNPs with iVLP assays. Here, for the first time, we established the pol I-based banyangvirus minigenome systems. By employing the pol I system combined with iVLP and superinfection assays, our study demonstrated the packaging of not only M segment by heterologous GP but also heterozygous RNPs by GP, further corroborating M segment reassortment potential. Moreover, besides M segments, L and S segments here were shown to be packaged by the structural proteins of heterogenous viruses to varying extents. These new insights further validate and characterize the genome reassortment potential of banyangviruses and some related phleboviruses, although which specific progeny viruses (especially those with L or S segment reassorted) could be generated and be viable need more detailed investigations by reverse genetics. By transient transfection, Rezelj et al. (2019) previously examined the minigenome activity of only M segment (but not L or S segments) driven by heterologous viral N and L which however, was shown to be quite low: SFTSV M minigenome activity driven by HRTV N and L was less than 2% and HRTV M minigenome activity driven by the replication proteins of SFTSV was less than 10%, compared to the respective M minigenome activity in the presence of cognate viral N and L. In contrast, we tested the minigenome activities of all the three segments by transfection, iVLP, and superinfection assays and intriguingly, the minigenome activities of M as well as L and S driven by the replication proteins of heterogenous viruses were much higher in all our experimental systems (transfection, iVLP, and superinfection): for instance, HRTV and SFTSV M minigenome activities driven by heterogenous replication proteins in the context of superinfections were respectively up to 77.2 and 68.7% of the corresponding activities driven by cognate viruses (Table 1), reflecting the greater reassortment potential. In addition, it was previously showed that SFTSV GP could not efficiently package HRTV L or S minigenomes, whereas the activity of HRTV GP in packaging SFTSV L minigenome seemed to be extremely high (even far higher than that of the cognate SFTSV GP itself) (Rezelj et al., 2019). However, in the present study, all the three segment minigenomes of HRTV could be high efficiently packaged by SFTSV GP, while packaging of minigenomes by GP of heterologous viruses was always reduced slightly or moderately, compared to that by cognate GP (Figure 4B). Aside from the different minigenome-generating systems, it should be noted that the minigenomes and structural proteins involved were from different viral strains. Here, we considered that the usage of different virus strains might be a reason for the different cross recognition activities (i.e., compatibility) observed, which may further imply that different virus strains may have different reassortment potential. It will be interesting to systematically compare inter-species or intra-species reassortment potential of various virus strains and particularly to further determine the strains having reassortment potential even stronger and hence meriting more attentions.

Despite the significant reassortment potential of SFTSV and HRTV, geographical isolation seems to reduce the risk of SFTSV-HRTV reassortment to a large extent. Following SFTSV, GTV is another banyangvirus recognized very recently in China and moreover, compared with HRTV, GTV is more closely related to SFTSV (Shen et al., 2018). Interestingly, we here demonstrated that SFTSV-driven minigenome activities of GTV were even stronger than those of HRTV (Figures 7D, 8), in line with their genetic relationships. Therefore, greater vigilance may be needed for the reassortment potential between SFTSV and GTV and close surveillance of potential co-prevalence of these viruses in China should be merited in future. In addition to the representative banyangviruses, several other important bunyaviruses which are less closely related to banyangviruses were also included in the present study as experimental controls. Intriguingly, although there is no minigenome activity of BUNV detected in the presence of SFTSV N and L, minigenome activities of RVFV and UUKV were significantly elicited by SFTSV protein expression or superinfection, albeit to lesser extents compared with those of GTV and HRTV. RVFV and UUKV are both representative phleboviruses; like SFTSV, RVFV is also a highly pathogenic virus, a potential bioterrorism agent, and a high-priority pathogen for research and development, while UUKV is non-pathogenic to humans (Schmaljohn and Nichol, 2007; Boshra et al., 2011; Word Health Organization [WHO], 2017). Although viral infectivity and pathogenicity should be affected by complex virus and host factors, many studies have suggested that the NSs proteins of these bunyaviruses are likely the major virulence determinant by manipulating host biological processes (Schmaljohn and Nichol, 2007; Hollidge Bradley et al., 2011). Indeed, NSs proteins of SFTSV, HRTV, and RVFV all have been identified by us and others as robust antagonists against host innate immunity despite functioning with different mechanisms, whereas the NSs of UUKV was suggested to be inefficient in immune antagonism (Hollidge Bradley et al., 2011; Ning et al., 2014, 2015, 2017, 2019; Rezelj et al., 2015; Kainulainen et al., 2016; Brennan et al., 2017). Based on the reassortment potential of the viruses shown in this study, genetic materials (including the potential virulence factors) from banyangviruses and at least some of the related phleboviruses may constitute gene pools, providing chances for emergence of novel pathogenic reassortants with highly genetic and phenotypic diversity.

UTRs of bunyaviruses contain *cis*-acting elements for transcription, replication, and packaging but vary in total length even for the same segment of different viruses (such as banyangviruses, as indicated in Figure 5) or different segments of the same virus (Prehaud et al., 1997; Flick et al., 2002, 2004; Kohl et al., 2004). However, the extreme terminal nucleotides of 5′ and 3′ UTRs which form the genome double-stranded panhandle by partial inverted complementarities are conserved among segments and viruses of the same genus. Consistent with previous studies on other bunyaviruses (Barr and Wertz, 2004; Flick et al., 2004; Kohl et al., 2004; Mir and Panganiban, 2006), our results here show that the panhandle structures of banyangviruses are also pivotal for viral UTR function and deletions of a conserved terminal region greatly deprived the minigenome activity. Moreover, the conserved panhandle region of banyangviruses cannot be functionally replaced by the corresponding terminal fragments of BUNV, suggesting the virus-specific requirement for the region. Further, a conservative protruding nucleotide within the panhandles of M and L (but not S) segments was identified as a new signature that contributes to the optimal UTR activity. Based on our results, this difference may partly lead to the higher promoter activity of M and L, compared to S. Although the conservative protruding nucleotide is mostly C, further characterization shows that it can be replaced by other bases (G, A, or U) without noticeable loss of minigenome activity. Nonetheless, the protruding nucleotide position in the conserved panhandle terminal region identified in this study seems to be important for its activity to positively regulate UTR function. The findings regarding the significant role of this protruding nucleotide are remarkable, as the protruding nucleotide, in some extent, decreases the panhandle complementarity which attracted major attentions of previous studies as the obvious feature needed for UTR activity (Barr and Wertz, 2004; Flick et al., 2004; Kohl et al., 2004; Mir and Panganiban, 2006). RNP formation, transcription, and replication are accomplished by complicated functional interactions of viral RNAs (especially UTRs), N, and L proteins, and thus this distinct signature of the panhandle may reflect some specific requirement within these interactions at least for banyangviruses. Further functional and structural studies on the replication machineries (including L, N, and the UTRs) and their delicate interactions will facilitate mechanistic elucidation on the positive modulation of RNP activity by the protruding nucleotide.

Additionally, not only homozygous but also heterozygous banyangvirus RNP function can be impaired by disruption of the protruding nucleotide (Figures 7D, 8, 9), suggesting that the nucleotide play roles in both banyangvirus replication and reassortment potential. Interestingly, the panhandles of some phleboviruses including the representative UUKV and RVFV also possess a conserved protruding nucleotide in M and L segments. Although it remains unclear whether the protruding nucleotide is required for homozygous phlebovirus RNP function, deletion of the nucleotide significantly reduced the minigenome activities driven by heterogenous replication machinery proteins from banyangvirus, suggesting that the protruding nucleotide is important for higher heterozygous RNP activity and thus stronger reassortment potential of banyangviruses with the related phleboviruses. On the other hand, introduction of the protruding nucleotide into HRTV S terminal panhandle that originally has no such nucleotide significantly enhances minigenome activity driven by not only cognate but also heterogenous virus superinfections. Therefore, such mutation of S segment could lead to enhancement of not only virus replication but also reassortment potential. In our study, superinfections with wild type viruses were comprehensively used in minigenome reporter assays, which corroborates viral reassortment potential and the notable roles of banyangviral panhandle region and the protruding nucleotide, whereas authentic reassorted or mutant progeny viruses were not generated, in consideration of biosafety. However, it may be merited to construct some point mutated viruses which are supposed to be attenuated for further unraveling the roles of the specific UTR features in virus replication and reassortment in the future.

In summary, the present study developed new pol I-based banyangvirus minigenome systems with luc or EGFP as readout reporters, which themselves may serve as valuable tools for elucidation of some virological mechanisms and high-throughput screening of antiviral drugs. Using the minigenome systems combined with transfection, iVLP, and superinfection assays, we provide evidence for remarkable reassortment potential among emerging banyangviruses as well as some related phleboviruses, highlighting the potential roles of reassortment in banyangvirus evolution and the necessity of continuing and close epidemiological surveillance on these emerging life-threatening viruses. Further, the protruding non-basepairing nucleotide in the genomic terminal panhandle was determined as a novel signature significant to optimal UTR activities driven by homogenous and heterogenous viral protein machineries. Identification and characterization of the protruding nucleotide not only shed lights on viral replication mechanism but also suggest that point mutations may further enhance (1) viral replication capability and hence pathogenicity and (2) viral structural element compatibility and thus reassortment potential. These findings provide new insights into viral replication and evolution mechanisms and may benefit the prevention and control of the emerging viral diseases in the future.

## Data Availability Statement

All datasets generated for this study are included in the article/Supplementary Material.

## Author Contributions

HW supervised the research. Y-JN conceived the study. Y-JN and FR designed the experiments. FR performed the experiments. FR, Y-JN, and HW analyzed the data. FD and MZ provided materials and contributed to completion of the study. Y-JN wrote the manuscript with some input from FR. All authors reviewed the results and approved the final manuscript version.

## Conflict of Interest

The authors declare that the research was conducted in the absence of any commercial or financial relationships that could be construed as a potential conflict of interest.
