# Supplementary material for: Combinatorial Minigenome Systems for Emerging Banyangviruses Reveal Viral Reassortment Potential and Importance of a Protruding Nucleotide in Genome “Panhandle” for Promoter Activity and Reassortment
Source: Front Microbiol. 2020 Apr 8;11:599. doi: 10.3389/fmicb.2020.00599 (PMC7156889; doi:10.3389/fmicb.2020.00599)
Supplement: Supplementary file 1 [file Data_Sheet_1.pdf]

Supplementary Data: Figure S1

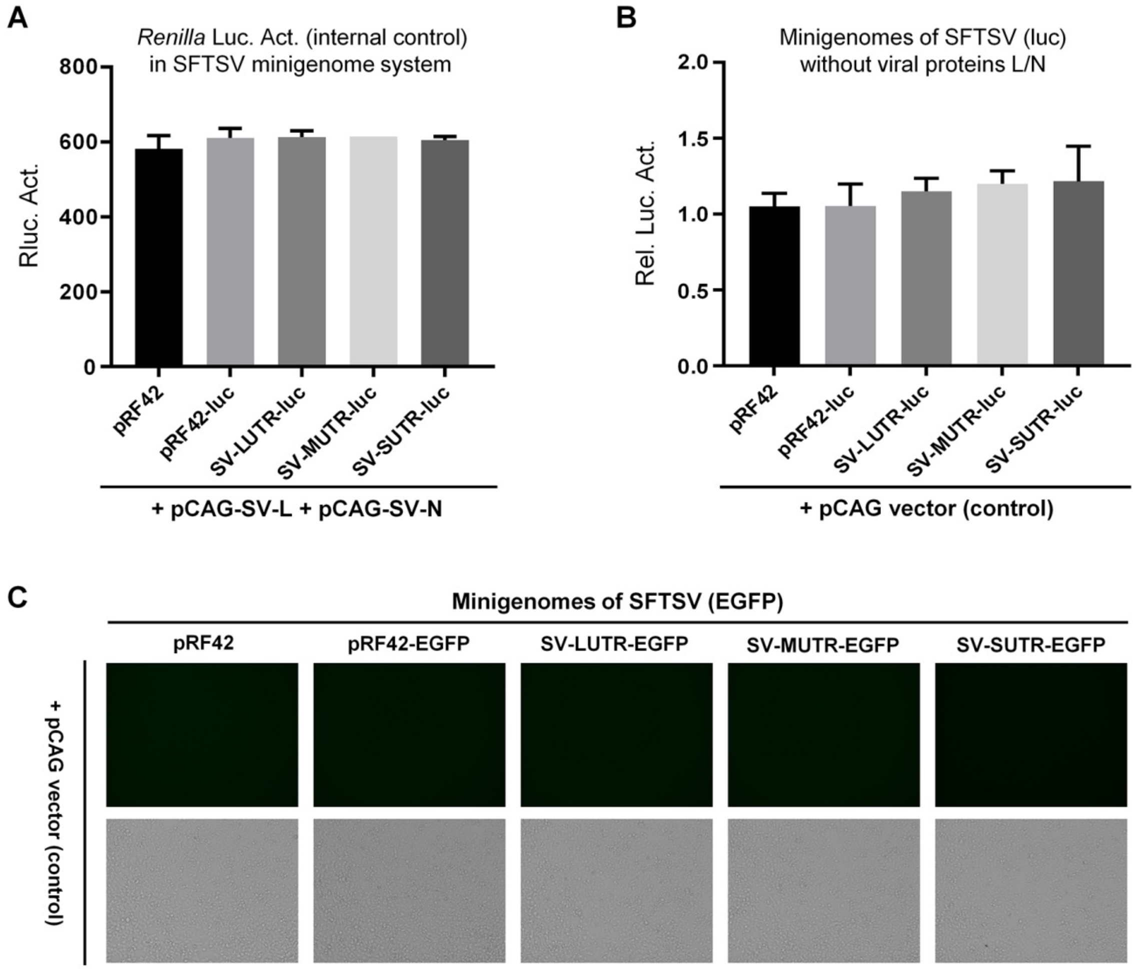

Figure S1. *Renilla* luciferase acitivities in the SFTSV minigenome systems in Figure 1B and SFTSV minigenome activities in absence of L and N. (A) The activities of *Renilla* luciferase (Rluc) expressed by the internal control plasmid (pRL-TK) cotransfected in the SFTSV minigenome systems in Figure 1B. Data are presented as mean ± SEM, $n = 3$. (B) SFTSV minigenome activities in luc reporter system without viral protein machineries, L and N. BHK-21 were transfected and treated as performed in Figure 1B, except that the viral protein expression plasmids (pCAG-SV-L and pCAG-SV-N) were replaced by the empty pCAGGSP7 vector (as control) during transfection. Forty-eight hours post-transfection, luc activities were measured. Data are means ± SEM, $n = 3$. (C) SFTSV minigenome activities in EGFP reporter system without L and N. Cells were transfected and observed as done in Figure 1C, except that the L and N expression plasmids were replaced by the empty pCAGGSP7 vector in the transfection assays.
